# Rapid Texture Optimization of Three-Dimensional Urban Model Based on Oblique Images

**DOI:** 10.3390/s17040911

**Published:** 2017-04-20

**Authors:** Weilong Zhang, Ming Li, Bingxuan Guo, Deren Li, Ge Guo

**Affiliations:** 1 State Key Laboratory of Information Engineering in Surveying Mapping and Remote Sensing, Wuhan University, Wuhan 430079, China; zhangweilong@whu.edu.cn (W.Z.); drli@whu.edu.cn (D.L.); 15872439113@163.com (G.G.); 2 Collaborative Innovation Center for Geospatial Technology, Wuhan University, Wuhan 430079, China

**Keywords:** oblique images, 3D texture reconstruction, radiation correction, occlusion detection, Graph-Cuts, texture mapping

## Abstract

Seamless texture mapping is one of the key technologies for photorealistic 3D texture reconstruction. In this paper, a method of rapid texture optimization of 3D urban reconstruction based on oblique images is proposed aiming at the existence of texture fragments, seams, and inconsistency of color in urban 3D texture mapping based on low-altitude oblique images. First, we explore implementing radiation correction on the experimental images with a radiation procession algorithm. Then, an efficient occlusion detection algorithm based on OpenGL is proposed according to the mapping relation between the terrain triangular mesh surface and the images to implement the occlusion detection of the visible texture on the triangular facets as well as create a list of visible images. Finally, a texture clustering algorithm is put forward based on Markov Random Field utilizing the inherent attributes of the images and solve the energy function minimization by Graph-Cuts. The experimental results display that the method is capable of decreasing the existence of texture fragments, seams, and inconsistency of color in the 3D texture model reconstruction.

## 1. Introduction

Low-altitude oblique images can be photographed from the side and the top of the city, which can not only provide a precise 3D surface model, but also obtain reliable texture information, becoming one of the principal data resources in urban 3D texture reconstruction and updating. Now, some reliable methods in the reconstruction of urban 3D surface models from oblique images have been proposed [1,2,3,4,5], especially for the commercialization of some software like Smart3D Capture. However, problems existed in low-altitude oblique images—such as great changes in field of view, large volume of data, inconsistency of light, perspective distortion, and occlusion—will the vast texture fragments and the discontinuity of color common, which needs to be solved. Research about these problems has been carried out in numerous of papers. Take the vertex rendering method epidemic in the early days, [6,7] assign the vertexes of the triangular by rear projection after color components of RGB of the aerial images. The texture generated lost great details with low resolution. References [8,9] utilized the method of weighting different images to generate texture, which did not work well with satisfactory consistency of the color of texture in many cases. In order to get a superior solution of these problems, many studies started to address how to choose the priority images as the texture resource for seamless texture mapping in a triangular mesh model. References [10,11] chose the priority image for each triangular facet as the texture resource by taking the visibility condition between the images and 3D surface model in to consideration. References [12,13] attempted to choose the highest quality image for every triangular facet as its texture after matching the corresponding line segments in the images and 3D surface model and making overall consideration of image occlusion, resolution, as well as the normal vector of the model. Reference [14] selected the priority texture for indoor small objects with close shots utilizing the Markov random field (MRF) method after overall optimization which obtained good results. Nevertheless, neither of these could address the problem of texture fragments and inconsistency of color in big urban-scene texture mapping. Moreover, the methods above require excessive computer resources resulting in low efficiency. On account of these problems, a method of rapid texture optimization will be presented in this paper to accelerate and improve the quality of seamless 3D urban texture reconstruction. In this paper, the experimental oblique images have been corrected for distortion, we will adopt a radiation procession algorithm to preprocess the oblique images first; and then, an efficient occlusion detection algorithm will be proposed for creating a priority list of visible images; at last, a Graph-Cuts based texture clustering and selecting algorithm will be put forward.

## 2. 3D Urban Surface Model

**Figure 1:**
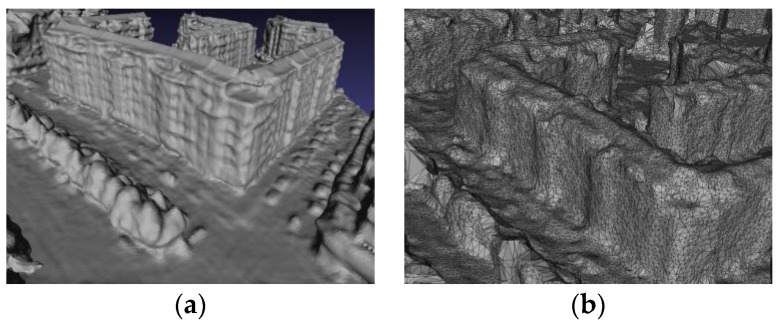
Local 3D model of the experimental area. (**a**) 3D fitting model; (**b**) 3D mesh model.

In the paper, the procedure of 3D urban terrain surface model reconstruction is as follows. First, extracting and matching feature points in the multi-view oblique images utilizing SIFT (Scale Invariant Feature Transform) [3] is combined with calculating the geometrical relationship between the multiple views. Then, the matching result by SFM (Structure from Motion) [1] is calculated to get scene information such as the camera position and then reconstruct sparse by the obtained 3D point cloud. After that, we combine the CMVS (Cluster Multi-View Stereo) and PMVS (Patch-based Multi-View Stereo) technique to acquire a reconstructed 3D dense point cloud [4,5]. The result is a constructed 3D urban surface model (expressed by Triangulated Irregular Network) for texture optimization and mapping. Figure 1 shows a local 3D model of the experimental area.

## 3. Image Radiation Preprocessing

On account of the time difference, environmental implications, and camera system error existing when shooting in multi-camera oblique photograph systems, there is a difference in brightness and color in different images shot on the same object. If the difference is left alone, it could result in color jump when reconstructing texture. Therefore, in this paper, a new algorithm is proposed which can correct the radiation inconsistency in multi-view images. In our algorithm, the local and overall environmental impacting factors are adjusted and the systemic error between and inside the camera is corrected, which could recover the mean value and the variance of the image to the initial radiation brightness value of the scene as far as possible and improve the brightness and color of the image used for texture extraction.

### 3.1. Correction of Environmental Radiation Error

The uniform color and light mechanism provided by Wallis filter is a relative radiation correction algorithm [15,16]. The algorithm can remove environmental radiation errors caused by global multi view by adjusting the mean value and variance of the image, which obtains a considerable result in uniform color and bright in aerial images. Formula (1) [15,16] is Wallis’ mapping function. However, during the processing of oblique photographs, on account of the vast gap of shooting distance on the oblique surface, a resultant effect of white mist appeared in some images caused by the inconsistency of atmospheric influence. As for the haze phenomena which has shrouded the cities of China for a long time; a superior result is not attainable by Wallis’ mapping function alone. Therefore, in this paper, the algorithm based on the dark channel in [17] is brought in to remove the phenomena of white mist caused by the inconsistency of depth. The experiment proved that the environmental radiation problem could be solved from the depth information of the images. Formula (2) [17] is the recovery function of the scene based on dark channel.

(1) $$f\left(x,y\right)=\left[g\left(x,y\right)-m_{g}\right]\frac{cs_{f}}{cs_{g}+\left(1-c\right)cs_{f}}+bm_{f}+\left(1-b\right)m_{g}$$

(2) $$J\left(x,y\right)=\frac{I\left(x,y\right)-A}{\max\left[t_{0},t\left(x,y\right)\right]}+A$$

In Formula (1), *f*(*x*,*y*) is the image after conversion and *g*(*x*,*y*) is the raw image. *s_f_*, *s_g_* and *m_f_*, *m_g_* are the standard deviation and mean value before and after conversion respectively. *c* ∈ [0, 1], which is the expansion constant of the variance of the image. *b* ∈ [0, 1], which is the brightness coefficient of the image. In Formula (2), *J*(*x*,*y*) is the image under ideal consideration; *A* is the intensity of atmospheric light; *t*(*x*,*y*) is the transmittance of transmission medium; *t*_0_ is the threshold of transmission.

### 3.2. Correction of Camera System Error

After the processing above in Section 3.1, the effect of environmental factor in different images is removed fundamentally, recovering their radiance to the level of a single image. Nevertheless, on account of the existing of camera system error, the phenomenon of the difference in brightness and color still appears in different images photographing the same object, which needs correcting. Generally, camera system error could be divided into exposure factor, white balance coefficient, camera response function, and the influence of halo. In the paper, other than the rest algorithms that only the camera response function taken into consideration to correct the camera system error, our proposed generalization camera response function including halo function, white balance coefficient, and exposure factor to correct the mapping from scene radiance to brightness value for color images. Formula (3) [18] is the mapping relation of the generalized camera response estimation function in the paper.

(3) $$\frac{g\left(B_{1}\right)}{w_{1i}e_{1}M\left(x_{1}\right)}=\frac{g\left(B_{2}\right)}{w_{2i}e_{2}M\left(x_{2}\right)}$$

In Formula (3), *g*(·) represents the inverse function of camera response function; *B* represents the brightness value of the image; *e* represents the exposure factor; *M* represents the halo function; *w_i_* represents the white balance coefficient in different channel; and *i* represents different channel.

## 4. Establishing a Visible Images List

Before the texture clustering and selection processed, it is required to know every triangular facet on the 3D surface model is visible in which images. Therefore, a list of visible images for each triangular facet in a 3D surface model needs to be established. In the paper, occlusion detection of triangular facets is implemented at first; then, according to the idea of texture filtering in [19], the occlusion area and normal vector are used as the evaluation criteria and a priority list of visible images is created.

### 4.1. OpenGL-Based Occlusion Detection

Different from the geometric model obtained by scanning a Lidar point cloud, it is uncertain to reconstruct all the surface features when utilizing oblique-image-based 3D reconstruction, which could lead to the inconsistency of occlusion between images and the 3D surface model. Therefore, algorithms of occlusion detection analogous to Z-buffer do not work well. In order to solve this problem, according to the transformation matrix of OpenGL in relation with the interior and exterior orientation elements in photogrammetry in [20], we completed the transformation matrix expression of OpenGL. Achieving rapid, efficient, and precise occlusion detection.

(4) $${\left[\begin{array}{ccc} u & v & 1 \end{array}\right]}^{\text{Τ}}=V_{3\times 4}\cdot P_{4\times 4}\cdot M_{4\times 4}{\left[\begin{array}{cccc} X & Y & Z & 1 \end{array}\right]}^{\text{Τ}}$$

**Figure 2:**
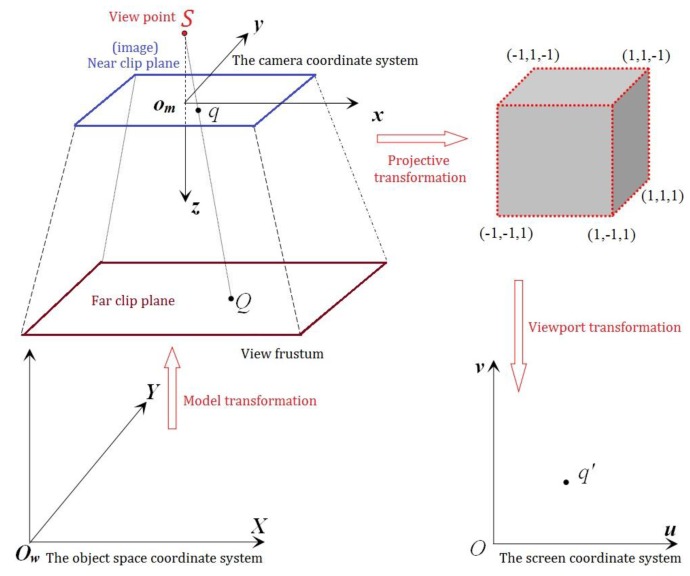
OpenGL transform procession.

Figure 2 shows three basic transformation of the procedure when OpenGL display 3D objects in window, they are model transformation (***M***), projection transformation (***P***), and viewport transformation (***V***). The object space coordinate system can be transformed into the screen coordinate system through these three transformation matrixes. The mathematical expressions of the matrixes are as seen in Formula (4). Formulas (5)–(7) are the specific expression of ***M***, ***P***, and ***V*** respectively, in which the model transformation equals the rotation and translation of the exterior orientation elements, *R* and *T_s_* represent the rotation matrix and translation matrix, respectively. Projection transformation equals to 3D cutting + similarity transformation + affine transformation, in which (*f*,*x*_0_,*y*_0_) is the interior orientation elements and (*w*,*h*) is the width and height of the camera. Viewport transformation is equal to 2D Euclidean transformation.

(5) $$M=\left[\begin{array}{cc} R^{T} & -R^{T}T_{s}\\ 0 & 1 \end{array}\right]$$

(6) $$P=\left[\begin{array}{cccc} \frac{2f}{w} & & -\frac{x_{0}}{w} & \\ & \frac{2f}{h} & -\frac{y_{0}}{h} & \\ & & -\frac{F+N}{F-N} & -\frac{2FN}{F-N}\\ & & -1 & \end{array}\right]$$

(7) $$V=\left[\begin{array}{cccc} \frac{w}{2} & 0 & 0 & \frac{w}{2}+x_{0}\\ 0 & \frac{h}{2} & 0 & \frac{h}{2}+y_{0}\\ 0 & 0 & 0 & 1 \end{array}\right]$$

### 4.2. Adaptive Partial Occluded Triangular Facet Subdivision

**Figure 3:**
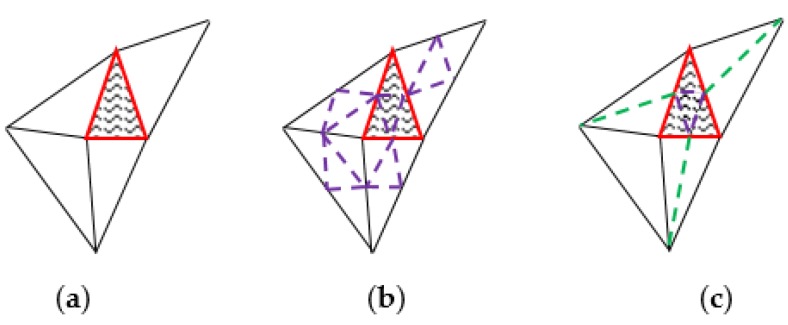
Triangle facet subdivision: (**a**) original mesh; (**b**) global quartering; (**c**) our method.

After occlusion detection, the images can be divided into three types for each triangular facet: fully occluded images, partially occluded images, and fully visible images. The fully occluded images are removed. However, if we do not process the triangular facets we only have partially occluded images, the texture inconsistencies might appear because of the 3D triangular mesh surface model subdivision is insufficient. In order to solve the problem and improve the occupancy of the non-occluded information in a single triangular facet, a local quartering algorithm is applied in adaptive partial occluded triangular facet subdivision in the paper. The new subdivision algorithm only partially occluded triangular facets and dichotomized the adjacent triangular facets (as Figure 3). In this way, not only the efficient of partial occluded triangular facets subdivision can be guaranteed, it can also decrease the time consumption and data size of segmenting non-occluded triangular facets. At the same time, it can also guarantee the invariability of half-edge data structure and the quality of manifold surfaces of the net form on the 3D surface model.

The shadow area in Figure 3 is the triangular facet which is partially occluded. Figure 3c shows the subdivision result of two different algorithms. Compared with the result of global quartering in Figure 3b, only in the worst situation, the number of triangular facets in Figure 3c is equal to Figure 3b. Generally, the amount of the triangular produced is 4*a* + 6*a* by our algorithm, which is smaller than 4*b*. *a* is the number of partially occluded triangular facets, *b* is the number of all of the triangular facets.

### 4.3. Priority Principle of Visible Images

**Figure 4:**
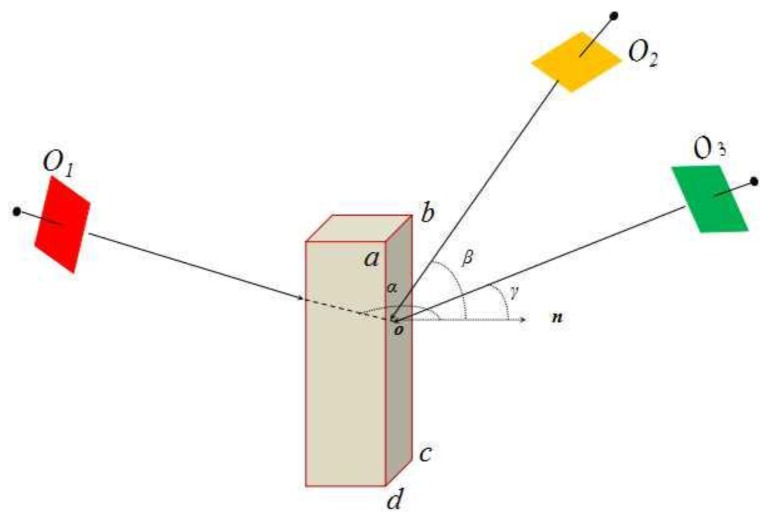
Occlusion conditions.

In the paper, we establish the visible images priority list following three principles below: (1) area maximum principle. That is to say, the larger area of the image on the triangular facet, the better; (2) angle minimum principle. In a non-occluded image, image deformation is proportional to the angle between photography light and terrain normal *n*. As shown in Figure 4, for surface *abcd*, image *O*_1_ is occlusion image, for *abcd* surface in the cube, image *O*_3_ has higher priority than image *O*_2_.

## 5. Texture Clustering and Selection Based on Graph-Cuts

Selecting the texture with the highest priority as the texture source for every triangular facet in the established priority list of visible images often cannot meet the global optimization condition. Therefore, how to choose the optimal image in the priority list of visible images as the texture mapping resource is still an intractable problem. In computer vision, a discrete multi-label problem like texture mapping can be regarded as a typical MRF model and Graph-Cuts is one of the most efficient global optimization tools to solve the energy function [21,22]. Thus, the Graph-Cuts based texture clustering algorithm under the frame of MRF is proposed to solve the problem of texture selection optimization in the paper.

### 5.1. Label Graph Construction

Graphing is an intuitive representation of the real word which is composed be a point set *V* and a set of sides connected between the nodes under a certain rule indicated as *G* = <*V*, *E*>. In this paper, each triangular facet in a 3D triangular mesh surface model is regarded as a node in graph *G*. Every two adjacent triangles with a common side respond an edge constructed between the nodes in graph *G*. In this way, we finish constructing the graph that we need. Graph-Cuts is a subset in set *E* of graph *G*. Graph-Cuts is a two-labeling problem, which could link the minimum of the energy function with the minimum cut of an image containing source and sink. As seen in the labeled graph constructed in this paper, not all the images could be the candidate texture resource to some certain triangular facets, as every triangular facet may be occluded in some image. That is to say, the mapping relation between triangular facets and images is not a complete graph, which needs to be processed.

**Figure 5:**
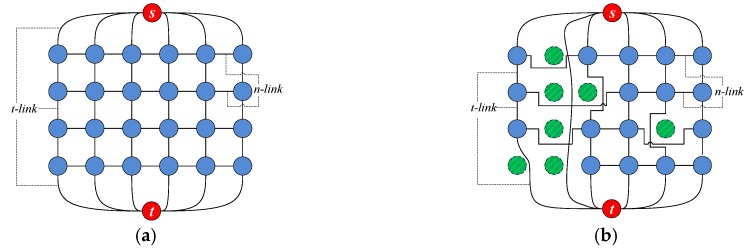
Labeled graph: (**a**) shows the general labeled graph; (**b**) shows our labeled graph.

As shown in Figure 5, Figure 5a is a general label graph, in which the top and bottom special red terminal nodes are the source *s* and sink *t* in the graph, respectively. The rest nodes are different labeled point to each triangular facet. The nodes are connected by side *t-link* and side *n-link* which is measured the probabilistic relation of Energy weight. *t-link* connects the sides of *s*,*t*, and other points which contains the energy between different labels. *n-link* connects the sides of middle points which contains the energy relation between adjacent nodes. The relations mainly reflect the probability of selecting the same texture label between the adjacent nodes. All the nodes constitute label space. Moreover, the surfaces in the same label layer have the same texture source. Figure 5b is the labeled graph constructed in the paper. Compared with Figure 5a, some green blank nodes appear, which means that texture nodes are undesirable in the texture space of this layer. Therefore, all the links between the nodes need to avoid the green blank nodes so that the data size of Graph-Cuts algorithm involved will be decreased and the overall efficiency of the algorithm can be improved.

### 5.2. Energy Function Construction

The energy function is the bridge between Graph-Cuts theory and a specific problem, it consists of two parts: data item and smooth item, which have one-to-one correspondence to weight of Graph-Cuts sides. The minimum cut in Graph-Cuts equals to the minimum energy function. Formula (8) is the functional expression of the energy function in the paper. In graph G, each node has a label set that contains k factors. The optimization algorithm selects the optimal label in the label set for each node. To guarantee calculation in the valid real range by the Graph-Cuts algorithm and the ability to process big data, the energy function construction needs to meet the following principles: (A) The energy function needs to possess upper and lower bounds; (B) The value range of the energy function needs to be no more than limit times of the number of the data calculated; (C) The data item, smoothed value, and fluctuating value of the energy are supposed to be in the same order of magnitude. In order to accurately reflect the identity of the corresponding texture resources in adjacent triangular facets, we constructed the data item and smooth item for energy functions using the inherent attributes of the candidate images, to achieve selecting optimization and clustering for textures in adjacent triangular facets.

(8) E(L)=αD(L)+βN(L)

(9) $$M=\left\{m_{i}|m_{i}=AVERAGE\left(I_{k}\right)\right\},i\in \left[0,n-1\right],k\in \left[0,O-1\right]$$

(10) $$D=\left\{d_{i}|d_{i}=STDEV\;\left(I_{k}\right)\right\},i\in \left[0,n-1\right],k\in \left[0,O-1\right]$$

(11) $$F=\left\{f_{i}|i\in \left[0,n-1\right]\right\}$$

(12) $$L=\left\{l_{i}|l_{i}\in \left[0,O-1\right],i\in \left[0,n-1\right]\right\}$$

In Formula (8), *α* and *β* are the control coefficients of the data item *D*(*L*) and smooth item *N*(*L*), respectively. Formulas (9–12) indicate the means value *M*, variance *D*, terrain triangular facet *F*, and the range of the label *L*, respectively. *O* is the number of the images and *n* is terrain surface. Formulas (13)–(14) are the expressions of the data item *D*(*L*) and smooth item *N*(*L*) in the energy function, in which *α_d_* and *β_d_* are internal factors of data items; *α_n_* and *β_n_* are internal factors of smooth item; *^V^f_i_l_i_* is the included angle between the terrain triangular surface *f_i_* and the responding image *l_i_*; *^W^f_i_l_i_* is the visual ratio, which is the ratio of visible area and real area.

(13) $$D\left(L\right)=\sum D_{P}=\sum \exp\left[1-P_{f_{i}}\left(l_{i}\right)\right]-1,\;P_{f_{i}}\left(l_{i}\right)=\left(\frac{\alpha_{d}\left(\pi /2-v_{f_{i}l_{i}}\right)+\beta_{d}w_{f_{i}l_{i}}}{\sum_{k\in \left[0,n-1\right]}\left[\alpha_{d}\left(\pi /2-v_{f_{k}l_{k}}\right)+\beta_{d}w_{f_{k}l_{k}}\right]}\right)$$

(14) $$\{\begin{array}{l} N\left(L\right)=\sum_{\left\{p,q\right\}\in N}N_{\langle p,q\rangle }\cdot \delta \left(l_{p},l_{q}\right),\;\delta \left(l_{p},l_{q}\right)=\{\begin{array}{ll} 0 & l_{p}=l_{q}\\ 1 & l_{p}\neq l_{q} \end{array}\\ N_{\langle p,q\rangle }=\exp\left(\min\left(\max\left(sqrt\left(\alpha_{n}\frac{{\left(m_{p}-m_{q}\right)}^{2}}{2\sigma_{1}^{2}}+\beta_{n}\frac{{\left(d_{p}-d_{q}\right)}^{2}}{2\sigma_{2}^{2}}\right)\right),n\_t\right)\right)-1 \end{array}$$

The energy function constructed in the paper satisfies not only the basic energy construction principle but also the normal distribution. Part of the parameter values in the formula above are as follows: *α* = 0.8, *β* = 0.2; *α_d_* = 0.8, *β_d_* = 0.2; *α_n_* = *β_n_* = 0.5. The parameters control the mean value and variance of the image directly. The *n*_*t* is the threshold value of the smooth item and here *n*_*t* = 1.

### 5.3. Energy Minimization Solution

In the paper, *α-β swap* optimization algorithm is utilized to solve energy function, which is an efficient Graph-Cuts based dichotomy optimization. Not only can the initial data set be optimized and segmented, it can also transform the multidimensional digraph to 2D simple digraph which can avoid bounded uncertainty [21,22,23]. The basic idea of the algorithm supposes that there is a known label set *L* and segmented set *P*. If *α*, *β* ∈ *L*, exchange the label set *α* and *β* to form a new label set *L_new_*. To make sure that the cut corresponding to the Graph-Cuts in the new label set is smaller than the original ones, the new label set is assigned to *L*. The procession is circulated in turn until the minimum cut of the Graph-Cuts appears. Any label set *L* to segmenting set *P* can be equal to *P* = {*P_l_*|*l* ∈ *L*} with no difference. *L* is the definitional domain of the label set. *P_l_* = {*p* ∈ *P*|*l_p_* = *l*} is the subset label of *l* which is a label in set *P*. Given a set of labels *α* and *β*, *α-β* exchange is applied. That is to say, exchange *α* and *β* to form a new segmented set *P_new_*, in which the set *l* ≠ *α*, *β* remains unchanged.

**Figure 6:**
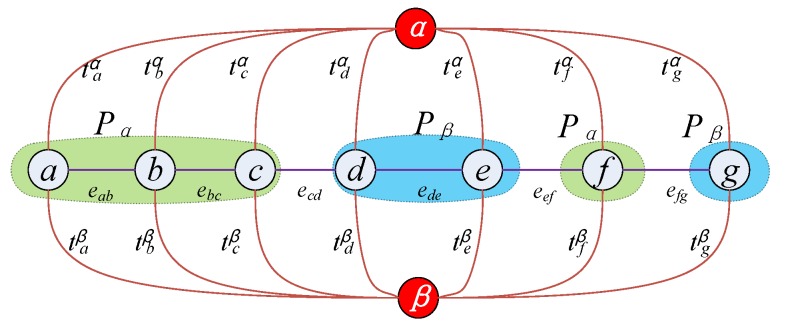
The *α*-*β* graph.

Figure 6 shows the construction way of *α-β* graph. *α* and *β* are connected through the *P_α_* set and *P_β_* set in the *α-β* graph, where *P_αβ_* = *P_α_*∪*P_β_* to define the weighted value of the corresponding *t-link* and *n-link*. *N_p_* is the neighborhood of node *p*, and the dissimilarity degree is labeled by *D_p_*(·), it guides the energy function space of Graph-Cuts; *V(·)* is the diversity measure of the neighborhood nodes, it has a smoothing effect.

## 6. Experiments and Analysis

### 6.1. Experimental Data and Environment

**Figure 7:**
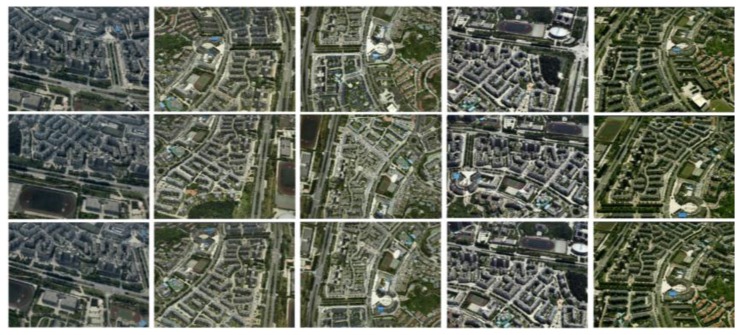
Experimental oblique images.

In order to objectively evaluate and verify the validity and superiority of the proposed method in the paper, we chose the real oblique images of an experimental area to test the method. The experimental images acquisition platform is SWDC-5, which consists of an upright camera and four cameras in the four directions in a 45° angle layout. The experimental computer environment is Windows 7 operating system, with 32 G computer memory, intel core 7 (3.6 GHz, four cores and eight threads). Figure 7 shows the five-view images of the experimental area.

### 6.2. Pyramid Design of PagedLoad

**Figure 8:**
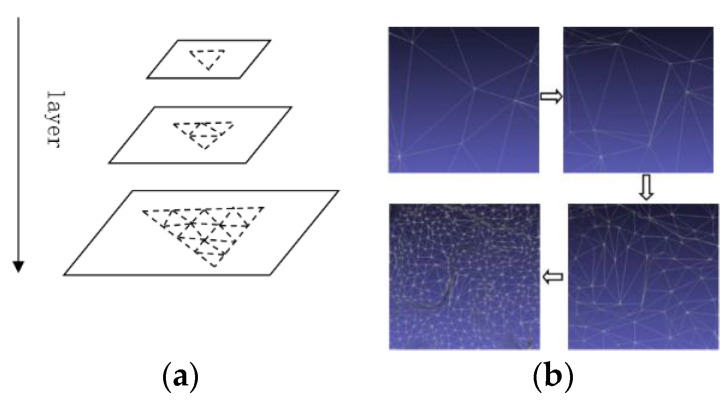
Mesh simplification. (**a**) Pyramid simplified model; (**b**) Scene simplified instance.

Large-scale urban 3D texture model reconstruction requires a lot of computing resources and storage space. In order to alleviate the limitations and conflicts of computer hardware and massive data, we designed a ready-to-use *PageLoad* pyramid model during the execution of the algorithm. The main idea of the *PageLoad* pyramid model is to layer and downsample the reconstruction scene according to the different visual distances and ranges of the observer to the scene. As shown in Figure 8, the grid of the top of the pyramid is the simplest, and the lower layers of the pyramid become more and more complex.

### 6.3. Results Analysis

#### 6.3.1. Comparison of Image Radiometric Correction

**Figure 9:**
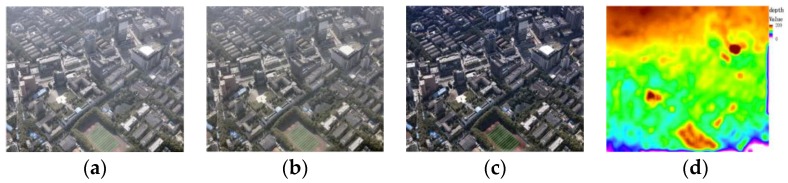
Contrast of defogging effect. (**a**) Original image; (**b**) Wallis processing result; (**c**) Dark channel processing result; (**d**) Depth map.

Figure 9 shows the defog effect using dark channels. From left to right in order are the original oblique image, Wallis processing result, dark channel processing result, and depth map estimated by dark channel. It can be easy seen from Figure 9 that the dark channel method has a better defog effect than the Wallis method, which can significantly enhance the contrast and color saturation of the image, and because the dark channel method can estimate the approximate depth range of the original image, we can achieve the desired defog effect without additional depth information.

**Figure 10:**
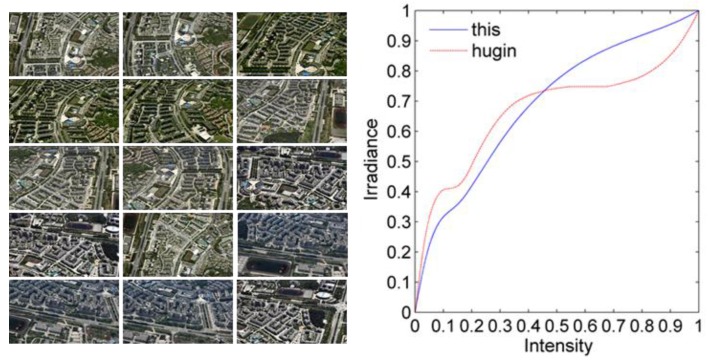
Contrast of camera response curves.

Figure 10 shows the comparison of the authenticity of our proposed method and Hugin [18] for the camera response curve estimation. The test data set is composed of the experimental oblique images in this paper. There are many ladder-like fluctuations in the camera response curve estimated by Hugin, the main reason is because it did not constrain the local constant function, that leads to a deformed camera response curve.

Since the Wallis has a good global radiometric correction effect in the absence of fog, and the dark channel algorithm is only applied to single image defogging, and the overall image is darker, therefore, in order to avoid the processed image still displaying radiation inconsistency after dark channel algorithm correction and our camera response function. The experimental oblique images are processed with the camera response function and the dark channel algorithm, and then Wallis processing, to make up for the defects of the dark channel algorithm without image-related information.

**Figure 11:**
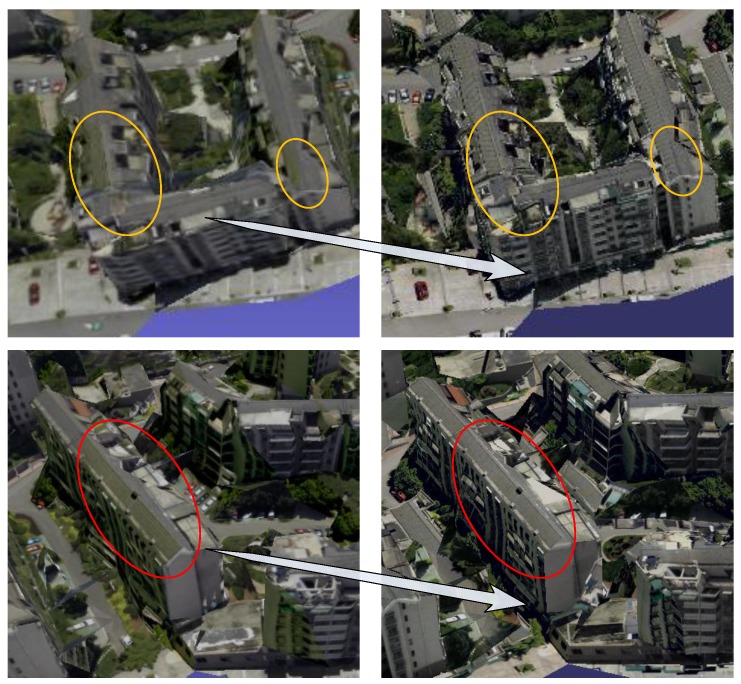
Contrast of texture radiation correction.

Figure 11 shows the local effects contrast of the 3D texture reconstruction before and after the radiation pretreatment. The left side of Figure 11 shows the effect of radiation pretreatment without our method; the right side are ours, where we can see that there is no texture color jump problem. After pretreatment of our radiometric correction, mapping textures not only maintain the high contrast of the image, but also the image saturation and white balance have been significantly adjusted, and the visual effect of texture model is more natural and clear.

#### 6.3.2. Efficiency Analysis of Occlusion Detection

**Figure 12:**
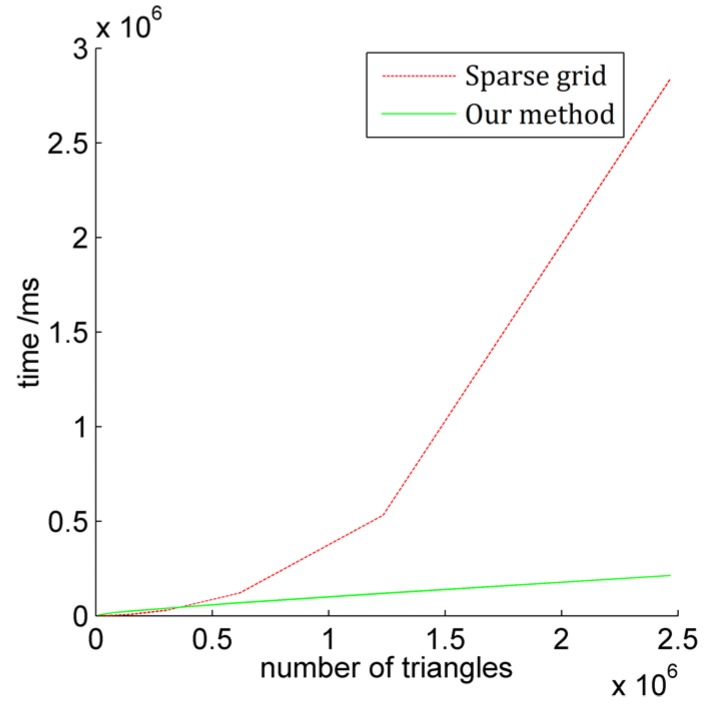
Efficiency contrast of occlusion detection.

Concerning occlusion detection efficiency, our use of the occlusion detection algorithm of Sparse Grid is based on Z-Buffer [24] as the reference object. The time complexity of traditional Z-Buffer is O(n^m^), the improved algorithm of Sparse Grid can be reduced to O(n), where m is the number of average pixels corresponding to each triangle facet, and n is the number of triangle facets. For the data set of massive triangular facets in a large-scale city scene 3D texture reconstruction, the spatial complexity of the improved algorithm mentioned above is non-uniform linear growth. Figure 12 shows the comparison result of the Sparse Grid algorithm and our occlusion detection algorithm proposed in the paper. It can be seen from Figure 12 that although the algorithm is not dominant when the amount of data is small, due to the optimization of OpenGL off-screen rendering detection mechanism and GPU acceleration, the linear steady growth of processing time can be ensured with by increasing the number of triangular facets. When the number of triangular facets is more than 300,000, the time consumption of the algorithm based on Sparse Grid shows abnormal jump growth with an increasing number of triangular facets, which demonstrates that our proposed occlusion detection algorithm has a significant advantage in dealing with large data volumes.

**Figure 13:**
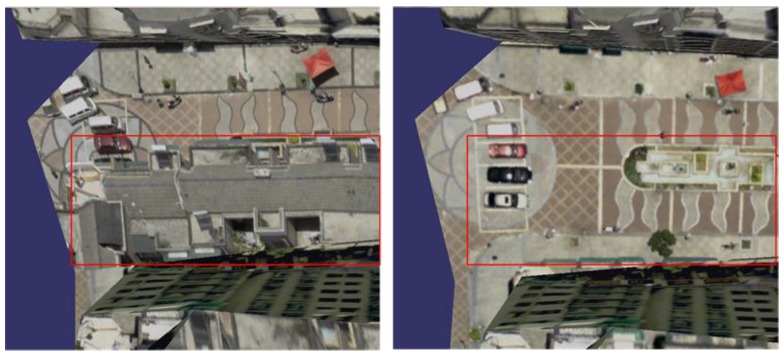
Results comparison of occlusion detection.

Figure 13 compares the effects of the occlusion detection. It can be seen from the red boxes that the occlusion phenomenon has been effectively removed in the 3D texture model on the right, guaranteeing the texture disparity coherence and consistency of the reconstructed 3D texture model.

#### 6.3.3. Comparison of Texture Clustering and Selection

**Figure 14:**
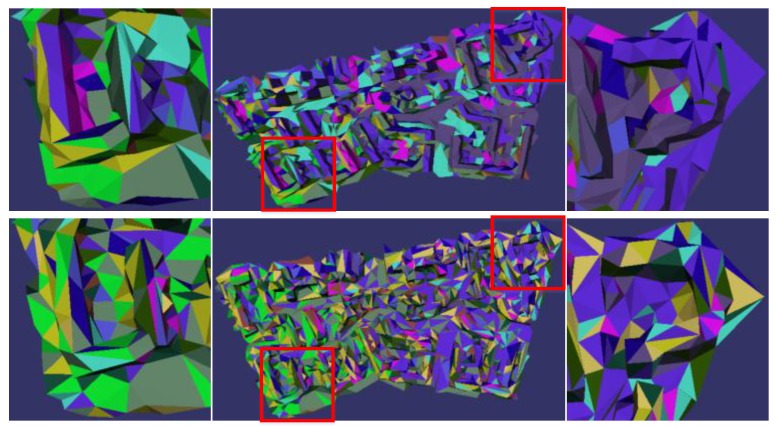
Contrast of clustering optimization.

Figure 14 shows the effects of texture clustering and selection, the above is the effect of our algorithm, the below effect is not optimal. The zoom view of four corners are the enlarged pictures corresponding to red frame, where the different colors represent the triangular texture taken from the different images. The comparison of the effects in Figure 14 is obvious, after texture clustering optimization, more adjacent triangular facets in the 3D triangular mesh surface model are given the same texture source, which can reduce the problem of texture fragments and seams. Besides, the results can also be used in the texture mapping operation to improve the efficiency of texture mapping by making the same clustering texture block and to obtain the corresponding textures at one time.

**Figure 15:**
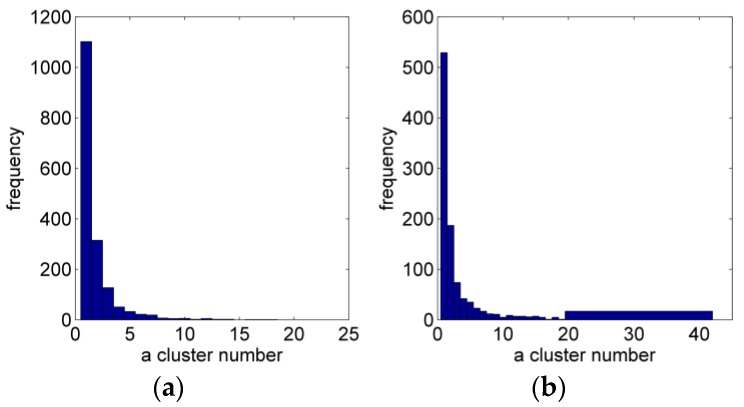
Comparison of the statistical results of clustering: (**a**) Before clustering; (**b**) After clustering.

Figure 15 shows the clustering statistic results of the triangular facets in the 3D triangular mesh surface model (3117 triangular facets). Figure 15a shows the texture selection distribution before clustering, Figure 15b is the result after clustering. According to the specific statistical data of clustering results, before clustering and selection optimization the isolated clustering (the number of adjacent triangular facets selecting the same texture source is 1 called isolated clustering) is about 35% of the total number of triangular facets, and the maximum clustering capacity is less than 20. After optimization, the isolated clustering is reduced to 15%, and the maximum clustering capacity is more than 40, which shows our optimization algorithm has a significant effect.

Table 1 is the efficiency comparison of energy minimization by Graph-Cuts with four different simplification 3D triangular mesh surface models. It can be seen from Table 1 that the processing time is directly proportional to the number of triangular facets, and it can converge with the global minimization quickly, the number of iterations is around 5, meanwhile, the adjacent iterative energy shows a decreasing trend. It should be noted that the local energy minimum value perhaps appears because the observed value is insufficient when the data set of the triangular facets is small. However, our constructed energy function can skip the local minimum value, and finally obtains the global minimization. This shows that our algorithm is stable and has high convergence efficiency and strong robustness.

#### 6.3.4. Effect Analysis of 3D Texture Reconstruction

**Figure 16:**
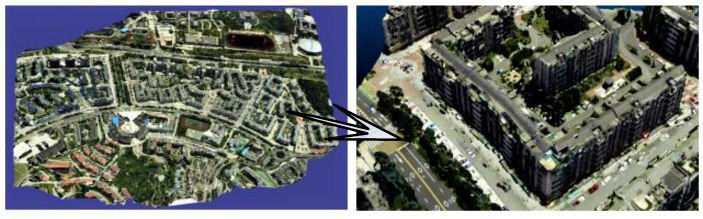
Texture reconstruction results.

**Figure 17:**
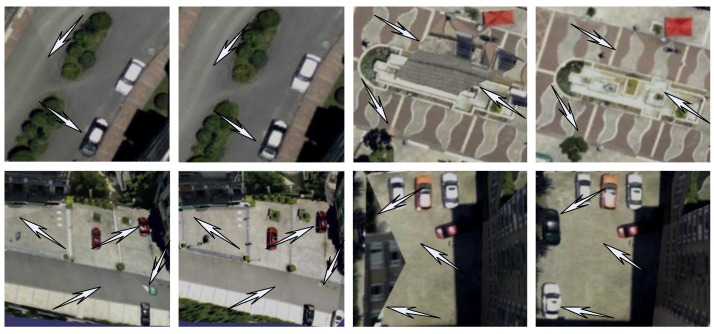
Contrast of local zoom views of texture optimization.

Figure 16 shows the experimental results of 3D texture reconstruction of our proposed method. The left side of Figure 16 is the global view of the 3D texture model in the experimental area, the right side of Figure 16 is the zoom view of the local 3D texture model. We can easily see the experimental results are better to maintain the consistency of color, realistic effects, and rich details. Furthermore, in order to more intuitively demonstrate the effectiveness and superiority of the proposed method, our method is compared with the famous 3D reconstruction software Photomodeler. As shown in Figure 17, there is some local amplification of the 3D texture model reconstructed by Photomodeler and our proposed method. From the areas indicated by the arrows in Figure 17, we can see that there are lots of texture inconsistencies, mapping errors, and seams in the results which are reconstructed by Photomodeler. After our texture optimization, the problems such as texture fragments and texture seams are significantly reduced, and the texture color continuity and integrity have been well maintained. 

## 7. Conclusions

In this paper, the experimental images are obtained by oblique photography technology which is used for 3D surface model reconstruction and as a texture extraction source. The main innovations are as follows: with respect to image radiation correction, the camera response function is introduced to deal with image reprocessing; with respect to occlusion detection, an efficient OpenGL detection method and a partial quartering algorithm are proposed; with respect to texture extraction, the discrete multi-label problem is solved by Graph-Cuts under the frame of MRF in texture selection optimization. Through the objective comparison of experimental results, the novel method of 3D urban texture model reconstruction is proved effective; can improve clustering expression and radiation consistency of texture mapping; and demonstrates advantages of high efficiency, fine automation level, realistic effects, and low cost.

## Figures and Tables

**Table 1:** Efficiency comparison of energy minimization by Graph-Cuts.

The Number of Triangular Facets	Convergent Rate of Energy Function	Number of Iterations	Energy Difference of Adjacent Iteration
3117	7.678 s	5	58.4, 5.8, 6.7, 4.2
7643	12.488 s	6	97.2, 68.4, 30.0, 3.6, 1.0
32,522	35.553 s	5	351.1, 66.8, 58.5, 20.2
136,834	162.729 s	4	1178.2, 278.3, 36.3
